# Concomitant Tumor Expression of EGFR and TATI/SPINK1 Associates with Better Prognosis in Colorectal Cancer

**DOI:** 10.1371/journal.pone.0076906

**Published:** 2013-10-25

**Authors:** Selja Koskensalo, Johanna Louhimo, Jaana Hagström, Mikael Lundin, Ulf-Håkan Stenman, Caj Haglund

**Affiliations:** 1 Department of Surgery, Helsinki University Central Hospital, Helsinki, Finland; 2 Department of Pathology, Haartman Institute, University of Helsinki, Helsinki, Finland; 3 Finnish Institution of Molecular Medicine Helsinki, University of Helsinki, Helsinki, Finland; 4 Department of Clinical Chemistry, Helsinki University Central Hospital, Helsinki, Finland; 5 Research Programs Unit, Translational Cancer Biology, University of Helsinki, Helsinki, Finland; Yokohama City University School of Medicine, Japan

## Abstract

**Background:**

Epidermal growth factor receptor (EGFR) activation plays a role in colorectal cancer (CRC) carcinogenesis, and anti-EGFR drugs are used in treatment of advanced CRC. One of the EGFR ligands is tumor-associated trypsinogen inhibitor TATI, also called serine protease inhibitor Kazal type1 (SPINK 1), which we recently showed to be an independent prognostic marker in CRC.

**Methods:**

We studied the prognostic value of immunohistochemical expression of EGFR and concomitant expression of EGFR and TATI/SPINK1 in a series of 619 colorectal cancer patients.

**Results:**

Of the samples, 92% were positive for EGFR. EGFR+/TATI+ was seen in 62.8%, EGFR+/TATI− in 29.5%, EGFR−/TATI+ in 4.9%, and EGFR−/TATI− in 2.7% of patients. EGFR expression correlated with WHO grade (p = 0.040). In univariate analysis, EGFR expression correlated with favourable survival (p = 0.006). EGFR+/TATI+ patients showed better survival than did those with other combinations (p<0.001). In multivariate analysis, EGFR+/TATI+ was an independent prognostic factor of favourable prognosis (p<0.001).

**Conclusion:**

Concomitant positivity of EGFR and TATI/SPINK1 predicts favourable prognosis in CRC.

## Introduction

Colorectal cancer (CRC) is the world's third most common malignancy [1],[2]. In Finland, the incidence is 30/100 000/year [3]. The most important prognostic factor in CRC is tumour stage.

Prognosis of patients with local CRC is good, 5-year survival being 80–90%, for node-positive tumors it is 60–70%, while for tumors with distant metastases it is less than 10%) [4]–[6]. Patients with stage III–IV (Dukes' C and D) disease usually receive adjuvant chemotherapy. In stage II (Dukes' B) disease, chemotherapy is not routinely used although some of these patients obviously would benefit from adjuvant therapy. To identify patients at high risk requires additional prognostic factors like biomarkers.

The epidermal growth factor receptor (EGFR), a target for treatment of advanced colorectal cancer, belongs to a transmembrane glycoprotein of the ErbB tyrosine kinase receptor family. Ligand-receptor interaction and dimerization of the receptor leads to tyrosine autophosphorylation, activating an intracellular signal pathway that promotes cell division and migration, inhibition of apoptosis, and angiogenesis [7]. The monoclonal antibodies cetuximab and panitumumab bind to EGFR and disable the activation of tyrosine-kinase and downstream signalling pathways.

Mutations in the molecular pathways activated via EGFR can contribute to carcinogenesis. In CRC, the most frequent mutations concern the KRAS gene occurs in about 40% of CRC cases [8],[9]. The mutations deactivate guanosine triphosphatase (GTPase) activity, leading to accumulation of activated KRAS. These KRAS mutations lead to lack of response to anti-EGFR therapy [10],[11].

Along with EGF, amphiregulin, transforming growth factor (TGF) α, epiregulin, betacellulin, heparin-binding EGF, and epigen activate EGFR [12]. Recently in pancreatic adenocarcinomas, serine protease inhibitor Kazal type1 (SPINK 1), also called pancreatic secretory trypsin inhibitor (PSTI) and tumour-associated trypsinogen inhibitor (TATI), was shown to activate EGFR [13]. TATI/SPINK1 is expressed together with EGFR in pancreatic adenocarcinomas. EGF and TATI/SPINK1 share about 50% amino acid homology [14], and the binding affinity of TATI/SPINK1 to EGFR is about half that of that of EGF [13].

We have recently shown that tissue expression of TATI/SPINK1 is an indicator of favourable prognosis in colorectal cancer patients [15]. In the present study we evaluated the relationship between EGFR and TATI expression and its possible prognostic value in colorectal cancer.

## Materials and Methods

### Patients

Clinical data were available from 643 consecutive patients who underwent surgery for histologically confirmed colorectal cancer at the Department of Surgery, Meilahti Hospital, Helsinki University Central Hospital, between 1982 and 1998. Complete clinical data and archival tissue specimens were available from 623 cases, 333 of them male. Median age was 65.9 years (range 22.7–90.3), and median follow-up time 4.81 years (range 0–25.8). Survival and cause of death data until March 2011 were obtained from the Population Register Centre of Finland, and Statistics Finland. Diagnosis and staging were performed according to the modified Dukes' classification [16]. The study has been approved by local ethics committee and complies with the Declaration of Helsinki (Dnro HUS 226/E6/06) and the National Supervisory Authority for Welfare and Health. Clinicopathological characteristics of the patients are described in Table 1.

**Table 1:** Patient clinicopathological characteristics and their correlation with EGFR immunoreactivity in 520 colorectal cancer patients assessed with chi-square test (^a^ Mann-Whitney test).

Clinicopathological	Patients	positive		*p* -value	
variable		(n)	%		
Gender				0.890	
Female	235	216	91.9		
Male	285	261	91.6		
Age				0.972	
<65 years	219	201	91.7		
≥65 years	301	276	91.7		
range 25.0–90.3, median 67.6 years				0.991	
Dukes' stage				0.145	
A	81	77	95.1		
B	188	177	94.1		
C	126	112	88.9		
D	125	111	88.8		
Dukes' stage				0.021*	
A and B	269	254	94.4		
C and D	251	223	88.8		
Differentiation (WHO grade)				0.040*	(Fishers')
1	17	16	94.1		
2	351	330	94.0		
3	130	113	86.9		
4	21	18	85.7		
missing	1				
Histologic type				0.244	
Adenocarcinoma	463	427	92.2		
Mucinous carcinoma	57	50	87.7		
Tumor location				0.948	
Colon	291	267	91.8		
Rectum	226	207	91.6		
missing	3				

* = p-value significant.

### Tissue samples and preparation of TMA blocks

Formalin-fixed and paraffin-embedded surgical tissue samples were collected from the archives of Department of Pathology, University of Helsinki. Histopathologically representative regions of tumour specimens were defined and marked on H&E slides. Three cores from each tumour block were sampled with 1.0 mm punchers by use of a semiautomatic tissue microarrayer (Tissue Arrayer 1, Beecher Instruments Inc., Silver Spring, MD, USA). Three parallel serial blocks were constructed, all including one sample from each patient. From each block, 4 µm thick sections were cut for immunohistochemistry.

### Immunohistochemistry

The Lab Vision Autostainer TM 480 (LabVision, Fremont, CA, USA) was used for immunostaining. Tissue sections were deparaffinized in xylene and rehydrated through graded alcohol series. To retrieve antigens, samples were heated in the pretreatment module of the autostainer in pre-heated TRIS-EDTA pH 9.0 buffer for 20 min at 98°C. The samples were incubated for 5 min in DAKO REAL Peroxidase–Blocking Solution (DAKO, Glostrup, Denmark) for inactivation of endogenous peroxidases. The sections were incubated for 60 min with primary monoclonal NCL-EGFR antibody (clone 113) against the extracellular domain, which stains both membrane and cytoplasma (Novo Castra, Newcastle, UK, dilution 1∶10). The sections were reacted for 30 min with HRP link and for 30 min with HRP enzyme (anti-mouse-anti-rabbit labelled polymer) (DAKO, Carpinteria, CA, USA). Between each of the steps the sections were rinsed with Tween 20-PBS (phosphate-buffered sodium chloride buffer, pH 9.0). Then the samples were incubated for 10 min in the DAB + EnVision System (DAKO, Glostrup, Denmark), and stained with hematoxylin for 1 minute. Finally the samples were rinsed in running tap water. Immunostaining of TATI was performed as described (Koskensalo 2011).

### Scoring

**Figure 1:**
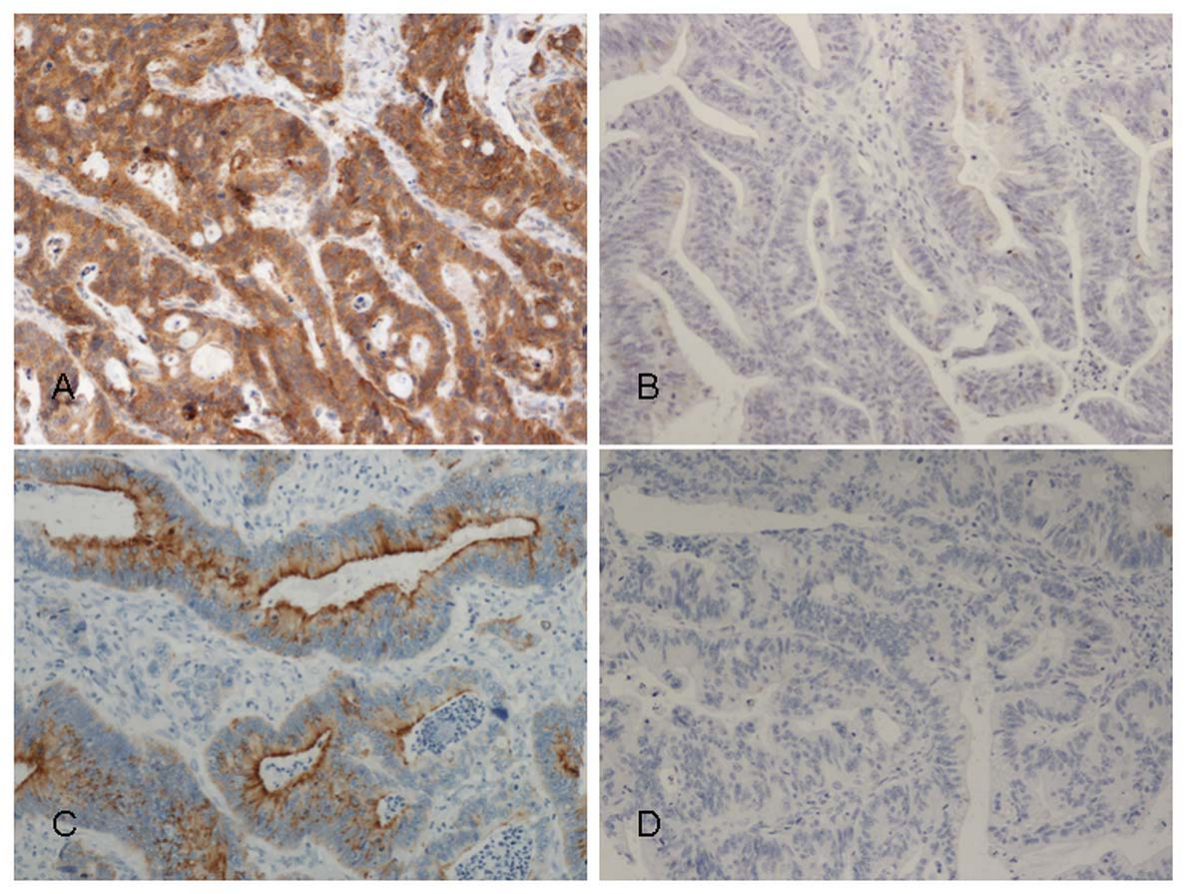
Immunohistochemical scoring pattern of EGFR and TATI/SPINK1 in colorectal cancer. A. EGFR positive, B. EGFR negative, C. TATI/SPINK1 positive, D. TATI/SPINK1 negative immunoexpression.

EGFR cytoplasmic immunopositivity in tumour cells was scored by two independent investigators (S.K. and J.H.) without knowledge of clinical outcome. Cytoplasmic EGFR immunopositivity was evaluated by percentage of positively stained cells. Positivity in over 50% of cells was scored as 3, 10–50% as 2, and less than 10% as 1 (Figure 1). Absence of positivity was scored as 0. Tissue spots without tumour cells were excluded. The highest score was used for each patient. For statistical analysis, the patients were divided into two groups: EGFR− (score 0) and EGFR+ (scores 1–3).

TATI/SPINK1 immunoexpression scoring was analogous to that of EGFR (Koskensalo 2011); for statistical analysis the patients were divided into two groups: TATI− (score 0) and TATI+ (scores 1–3).

### Statistical analysis

We analyzed separately EGFR staining alone and together with TATI/SPINK1 staining (EGFR+/−, TATI+/− or EGFR−TATI+/−). The association between immunoexpression and clinicopathological variables was assessed by the χ^2^ test or Fisher's exact test in case of low expected frequencies. Survival was analyzed by the Kaplan-Meier method. Statistically significant covariates in univariate analysis were analyzed in multivariate survival analysis by the Cox proportional hazards model. A p<0.05 was considered significant. Statistical analyses were performed with IBM SPSS Statistics 20.0 software.

## Results

### Immunostaining for EGFR

Reliable evaluation of immunostaining was possible in 520 of the 623 samples. In 69 (13.3%), cytoplasmic EGFR immunopositivity was scored as high (3), in 172 (33.1%) as moderate (2), and in 236 (45.4%) as weak (1), while 43 (8.3%) showed no EGFR immunopositivity (Fig. 1). We evaluated only cytoplasmic staining, because if cytoplasma is positive, the membrane staining is unsuitable for reliable evaluation.

EGFR immunoreactivity associated with tumour grade; positivity was detected more often in highly and moderately differentiated than in undifferentiated tumours (p = 0.040). EGFR expression showed no association with Dukes' stage, but positivity was more often present in local (Dukes' A–B) than in metastasized tumours (Dukes' C–D) (94.4% versus 88.8%, p = 0.021)(Table 1).

### Simultaneous immunostaining for EGFR and TATI/SPINK1

Evaluation of immunostaining for both EGFR and TATI/SPINK1 was possible in 511 of the 623 samples. The combination EGFR+/TATI+ was present in 321 (62.8%), EGFR+/TATI− in 151 (29.5%), EGFR−TATI+ in 25 (4.9%), and EGFR−/TATI− in 14 (2.7%) patients.

EGFR+/TATI+ correlated with histology occurring more often in adenocarcinomas than in other histological tumour types (p = 0.005), and varied by WHO grade, being most often present in highly and moderately differentiated tumours (p<0.001)(Table 2).

**Table 2:** Patient clinicopathological characteristics and their correlation with EGFR and TATI immunoreactivity in 511 colorectal cancer patients assessed with chi-square test

Clinicopathological variable	Patients	EGFR−TATI−		EGFR−TATI +		p	EGFR+TATI−		EGFR+TATI+		*p*
		(n)	%	(n)	%		(n)	%	(n)	%	
Gender						0.051					0.88
Female	232	9	3.9	8	3.4		71	30.6	144	62.1	
Male	279	5	1.8	17	6.1		80	28.7	177	63.4	
Age						0.546					0.55
<65 years	215	7	3.3	10	4.7		58	27.0	140	65.1	
≥65 years	296	7	2.4	15	5.1		93	31.4	181	61.1	
range 25.0–90.3, median 67.6 years											
Dukes' stage					0.482					0.21	
A	79	0	0	3	3.8		22	27.8	54	68.4	
B	185	3	1.6	7	3.8		51	27.6	124	67.0	
C	124	6	4.8	7	5.6		37	29.8	74	59.7	
D	123	5	4.1	8	6.5		41	33.3	69	56.1	
Dukes' stage					0.238					0.02	
A and B	264	3	1.1	10	3.8		73	27.7	178	67.4	
C and D	247	11	4.4	15	6.1		78	31.6	143	57.9	
Differentiation (WHO grade)				0.184					<0.001		
1	17	0	0	1	5.9		1	5.9	15	88.2	
2	347	4	1.2	14	4.0		86	24.8	243	70.0	
3	125	9	7.2	7	5.6		52	41.6	57	45.6	
4	21	1	4.8	2	9.5		12	57.1	6	28.6	
missing	1										
Histologic type					0.286					<0.005	
Adenocarcinoma	457	13	2.8	20	4.4		126	27.6	298	65.2	
Mucinous carcinoma	54	1	1.9	5	9.3		25	46.3	23	42.6	
Tumor location					0.546					0.76	
Colon	287	7	2.4	15	5.2		89	31.0	176	61.3	
Rectum	221	7	3.2	10	4.5		62	28.1	142	64.3	
missing	3										

### Prognostic value of EGFR

In univariate analysis, EGFR immunoexpression (p = 0.006), patient age (p = 0.009), WHO grade (p<0.001), and Dukes' stage (p<0.001) associated with prognosis. Five-year survival was 59.9% in EGFR+ patients and 40.5% in EGFR− patients (Table 3).

**Table 3:** Univariate analysis of correlations between preoperative characteristics and survival by Kaplan-Meier life-table and logrank test analyses.

Clinicopathological	Patients		Cumulative 5-y	χ2	*p*- value
variable	(n)	%	survival %	statistic	
Gender				0.002	0,964
Female	235	45.2	56.9		
Male	285	54.8	60.4		
Age				6.739	0.009*
<65 years	219	42.1	63.3		
≥65 years	301	57.9	55.5		
Dukes' stage				266.05	<0.001*
A	81	15.6	89.0		
B	188	36.2	83.1		
C	126	24.2	52.4		
D	125	24.0	8.2		
Dukes' stage				196.02	<0.001*
A and B	269	51.7	84.9		
C and D	251	48.3	30.7		
Differentiation (WHO grade)				12.93	<0.001*
1	17	3.3	80.9		
2	351	67.6	62.3		
3	130	25.0	49.7		
4	21	4.0	38.9		
missing	1				
Histologic type				0.751	0.386
adeno-ca	463	89.0	59.8		
mucinous ca	57	11.0	51.6		
				2.253	0.133
Tumor locationColon	291	56.3	60.7		
Rectum	226	43.7	56.8		
missing	3				
EGFR immunoreactivity				7.549	0.006
negative	43	8.3	40.5		
positive	477	91.7	59.9		

* = p-value significant.

In multivariate survival analysis, EGFR (p = 0.023), patients' age (p<0.001), Dukes' stage (p<0.001), tumour location (p = 0.001), and WHO grade (p = 0.033) were independent prognostic factors (Table 4).

**Table 4:** Cox multivariate regression analysis of prognostic factors in 520 colorectal cancer patients.

Covariate	Wald statistic	p-value	RH	95% CI
Age	31.006	<0.001	1.032	1.021–1.044
Dukes' stage	285.799	<0.001		
A				
B	0.519	0.471	1.259	0.673–2.357
C	29.923	<0.001	5.084	2.839–9.103
D	123.403	<0.001	27.601	15.371–49.562
WHO Grade	8.276	0.041		
1				
2	3.579	0.058	2.619	0.966–7.103
3	5.282	0.022	3.305	1.192–9.161
4	6.197	0.013	4.341	1.366–13.791
Tumor location in rectum	10.009	0.002	1.535	1.366–13.791
Histologic type		NS		
EGFR	4.054	0.044	0.639	0.413–0.988

NS = not significant, RH = relative hazard, CI = confidence interval at 95% level.

### Prognostic role of the combination of EGFR and TATI/SPINK1

**Figure 2:**
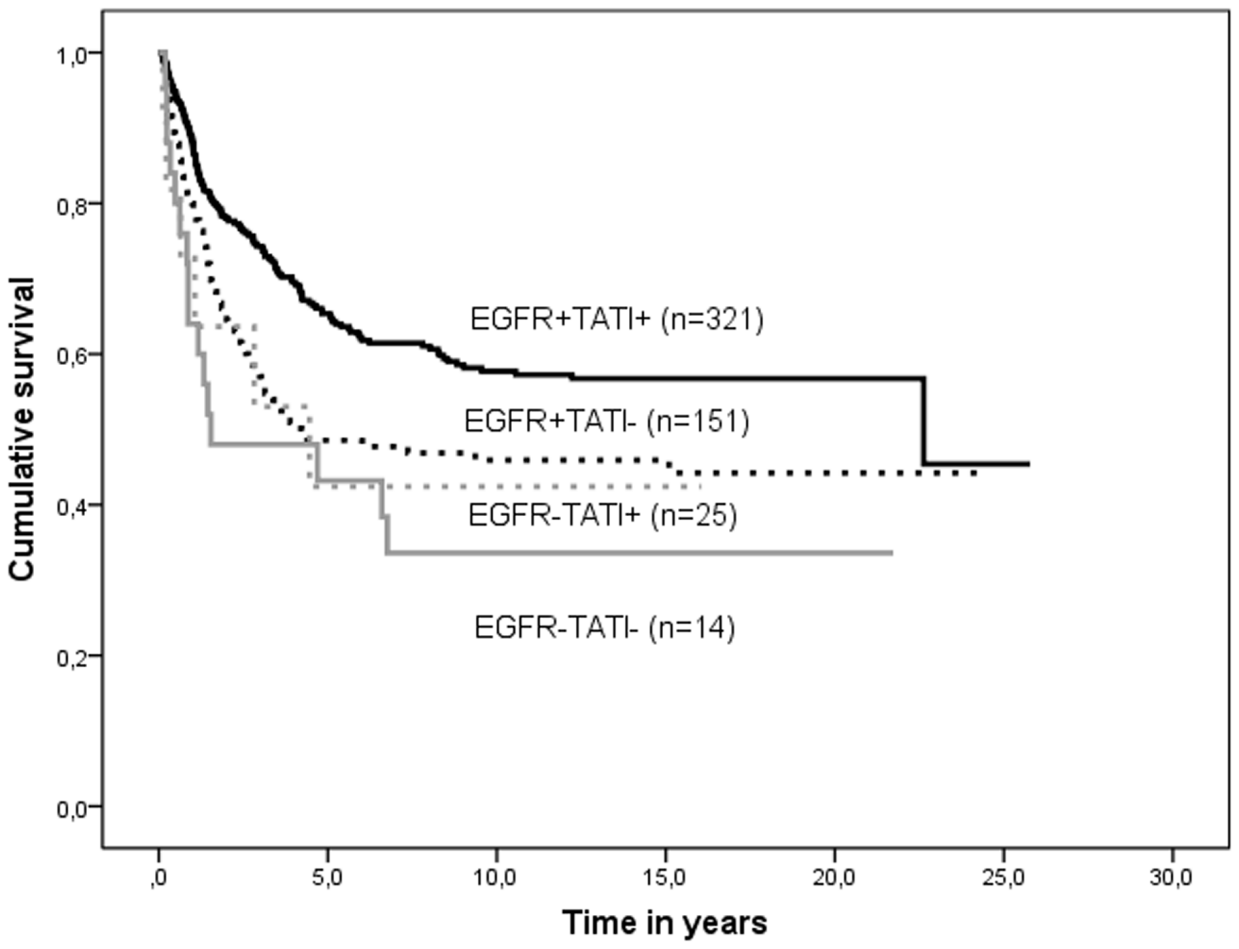
Survival curves of concomitant expression of EGFR and TATI/SPINK1 in colorectal cancer patients.

Concomitant expression of EGFR and TATI/SPINK1 correlated with prognosis, 5-year survival being 65.0% in EGFR+/TATI+ patients, 47.7% in EGFR+/TATI−, 43.2% in EGFR−/TATI+, and 42.4% in EGFR−/TATI− patients (p<0.001)(Table 4, Figure 2). High age (p = 0.009), advanced Dukes' stage (p<0.001), and advanced WHO grade (p<0.001) correlated with poor prognosis (Table 5).

**Table 5:** Univariate analysis of correlations between preoperative characteristics and survival with Kaplan-Meier life-table and logrank test analyses.

Clinicopathological	Patients		Cumulative 5-year	χ2	*p*-value
variable	(n)	%	survival %	statistic	
Immunoexpression				13.135	<0.001
EGFR+TATI+	321	62.8	65.4		
EGFR+TATI−	151	29.5	48.5		
EGFR−TATI+	25	4.9	43.2		
EGFR−TATI−	14	2.7	42.4		
Gender				0.002	0.964
Female	235	45.2	56.9		
Male	285	54.8	60.4		
Age				6.739	0.009
<65 years	219	42.1	63.3		
≥65 years	301	57.9	55.5		
Dukes' stage				266.05	<0.001*
A	81	15.6	89.0		
B	188	36.2	83.1		
C	126	24.2	52.4		
D	125	24.0	8.2		
Dukes' stage				196.02	<0.001*
A and B	269	51.7	84.9		
C and D	251	48.3	30.7		
WHO grade				12.93	<0.001*
1	17	3.3	80.9		
2	351	67.6	62.3		
3	130	25.0	49.7		
4	21	4.0	38.9		
missing	1				
Histologic type				0.751	0.386
adeno-ca	463	89.0	59.8		
mucinous ca	57	11.0	51.6		
Tumor location				2.253	0.133
Colon	291	56.3	60.7		
Rectum	226	43.7	56.8		
missing	3				

* = p-value significant.

In multivariate survival analysis, EGFR/TATI expression (p<0.001), age (p<0.001), Dukes' stage (p<0.001), and location (p = 0.003) were independent prognostic factors (Table 6).

**Table 6:** Cox multivariate regression analysis of prognostic factors in 511 colorectal cancer patients.

Covariate	Wald statistic	p-value	RH	95% CI
Age	29.817	<0.001	1.032	1.020–1.043
Dukes' stage	282.046	<0.001		
A				
B	0.921	0.337	1.371	0.720–2.612
C	32.723	<0.001	5.813	3.180–10.624
D	121.32	<0.001	30.23	16.482–55.445
Tumor location in rectum	8.812	0.003	1.499	1.148–1.959
EGFR+TATI+	20.691	<0.001		
EGFR+TATI−	14.556	<0.001	1.771	1.321–2.376
EGFR−TATI+	10.768	0.001	2.438	1.432–4.151
EGFR−TATI−	0.306	0.580	0.554	0.554–2.869
Differentiation (WHO Grade)	NS			
Histologic type		NS		

NS = not significant, RH = relative hazard, CI = confidence interval at 95% level.

## Discussion

We recently showed that TATI is an independent prognostic factor in colorectal cancer, high tissue expression being associated with favourable prognosis. Here we show that concomitant immunoexpression of EGFR and TATI is an independent prognostic factor for favorable survival in colorectal cancer, and it is a stronger prognostic factor than EGFR or TATI/SPINK1 alone.

In concordance with previous findings, we observed EGFR overexpression in colorectal cancer samples, [17],[18]. EGFR immunoexpression correlated significantly with tumour grade, being more often positive in high and moderately differentiated tumours, as reported previously [19]. Conversely, EGFR expression has also been shown to correlate with poor differentiation [18]. As in other studies, we found correlation with histology [20],[21] but no correlation between EGFR immunoexpression and Dukes' stage. This is in concordance with the study by Giralt [20], whereas Spano et al. reported stronger EGFR overexpression in T3 than in T4 colorectal tumours [17]. Deng et al. found, in 94 colorectal cancer patients, an association between high expression and high tumour stage [22].

Here we show that EGFR immunoexpression is an independent marker for favourable prognosis in colorectal cancer patients. Our results differ from those others' ; EGFR expression did not correlate with survival in a study of 249 [19] and 132 CRC patients [17] or in 87 Dukes' C CRC patients, [6]. However, in some studies specifically of colon cancer, EGFR expression correlated with poor prognosis. In a study of 149 patients, EGFR expression was an independent marker of adverse prognosis, but EGFR expression was observed in only 35.6% of the samples, which is less than usually reported [23]. Resnick et al. also showed an association between strong EGFR expression and poor prognosis in colon cancer [24]. In a subanalysis including only colon cancer, our study showed an association between EGFR expression and improved survival, but the difference was not significant (data not shown).

In a subgroup analysis of rectal cancer, we found significantly better survival rates (p = 0.001) in patients with EGFR positivity (data not shown). In 87 rectal cancer patients who had received preoperative radiation therapy, Giralt et al reported EGFR expression in pre-treatment biopsies- but not in surgical samples- to associate with adverse prognosis [20]. In a study by Fernebro et al. on 269 rectal cancer patients, no association was observed with metastasis-free survival [25]. In a study of 40 rectal cancer patients, Yasuda et al. found decreasing EGFR levels after chemoradiotherapy, but EGFR expression was not a prognostic marker [26]. Radiotherapy can both reduce and increase EGFR expression [27],[20]. We did not analyze EGFR expression in pre-treatment biopsies, but no difference in EGFR expression emerged between rectal cancer patients receiving or not receiving preoperative radiotherapy (data not shown).

EGFR-targeted treatments have been used for metastasized colorectal cancers, but the value of EGFR immunoexpression to predict the efficiency of adjuvant treatment is controversial. Interestingly, the EGFR antagonist cetuximab has proven effective even against EGFR-negative tumours [28]. One explanation for this may be that anti-EGFR- treatment is targeted against metastasized tumors, and correlation between EGFR immunoexpression in the primary tumor and metastatic lesion is unclear and varies between studies [19],[29],[22]. In addition, EGFR-positive tumors do not always respond to cetuximab treatment [30]. Furthermore, tumors with KRAS mutations have been found to respond poorly to anti-EGFR therapy [10],[11], and the analysis of KRAS mutational status is recommended for all metastatic CRCs [31]. KRAS mutations in codon 12 correlates with adverse prognosis [31], [32]. BRAF is the second step of the EGFR- mediated pathway. Mutated BRAF correlates with adverse prognosis [33], but patients with BRAF mutation may benefit from EGFR-targeted treatments [31]. A novel method that appears to be superior to analysis of KRAS mutations to select patients for anti-EGFR therapy is combined analysis of KRAS mutations and EGFR gene copy number [34].

The PIK3CA (the phosphatidylinositol-4,5-bisphosphonate 3-kinase, catalytic subunit alpha polypeptide gene) pathway is another downstream pathway of EGFR signalling. Mutations of the PIK3CA gene can promote malignant transformation [35]. Use of aspirin blocks the PIK3CA pathway, and correlates with better prognosis among patients with a mutated gene [36]. Unfortunately data on aspirin use was not available in our patient records.

Ozaki et al. showed that EGFR and TATI/SPINK1 are co-expressed in pancreatic cancer and that SPINK1 stimulates the proliferation of pancreatic cancer cells through EGFR activation [13]. Here we show that in roughly two out of three colorectal carcinoma samples EGFR and TATI/SPINK1 are co-expressed. We have shown earlier that high TATI/SPINK1 tissue expression correlates with better prognosis in CRC; here we show that concomitant expression of EGFR and TATI/SPINK1 is an even better marker for improved survival. It is known that there are positive and negative feedback loops within EGFR-mediated pathways depending on cell type, and one reason for better prognosis in colorectal cancer may be that binding of TATI leads to inhibition of cascades leading to malignant transformation. Also, TATI binding to EGFR may inhibit the binding of stronger activating ligands.

It is therefore plausible that TATI/SPINK1 and the interaction between TATI/SPINK1 and EGFR play different roles in CRC than in pancreatic cancer.Interestingly, expression of TATI/SPINK1 in cancer tissue is also associated with good prognosis in gastric cancer [37] and loss of TATI/SPINK1 expression correlates with aggressive disease in bladder cancer [38]. This indicates that TATI/SPINK1 exerts different functions in different pathologies. TATI/SPINK1 is not only an activator of EGFR but also an effective trypsin inhibitor and trypsin is expressed by several tumors [38]. Thus it is not surprising if it also exerts different functions in different tumours. So far, EGFR immunohistochemistry is not a tool for prediction of which patients that are likely to benefit from EGFR-targeted adjuvant treatment. It would be interesting to study whether adding TATI/SPINK1 tissue analysis plus KRAS mutation analysis and EGFR gene copy number assessment could further improve the promising results of Ålgars et al. [34].

In conclusion, to the best of our knowledge, we show for the first time that concomitant immunoexpression of EGFR and TATI/SPINK1 is an independent prognostic marker for favourable prognosis in colorectal cancer patients. The combination is a stronger prognostic tool than either TATI/SPINK1 or EGFR alone. Further studies are necessary to better understand the association between TATI/SPINK1 and EGFR in colorectal cancer and for evaluating the potential use of this marker combination to predict treatment response.

## References

[1] Boyle P, Levin B (2008). World Cancer Report. IARC: Lyon, France.

[2] Ferlay J, Bray F, Pisani P, Parkin M (2004). Cancer insidence, Mortality and Prevalence Wold wide. Globocan 2002. IARC CancerBase No. 5. IARC Lyon.

[3] Finish Cancer Registry. http://www.cancer.fi/syoparekisteri/en/statistics/cancer-statistics/ Accessed 2013 March 19.

[4] Howlader N, Noone AM, Krapcho M, Neyman N, Aminou R, et al.. (2011). SEER Cancer Statistics Review, 1975–2008, National Cancer Institute. Bethesda, MD.

[5] Stephen BE BD, Compton CC, Fritz AG, Greene FL, Trotti A (ed) (2010) AJCC. Part III Digestive System: Colon and Rectum. In Cancer Staging Handbook, Seventh edition edn, Chapter Part III Di gestive System, pp 173–206. New York: Springer.

[6] Cunningham MP, Essapen S, Thomas H, Green M, Lovell DP, et al (2006) Coexpression of the IGF-IR, EGFR and HER-2 is common in colorectal cancer patients. Int J Oncol 28: 329–335. 16391786

[7] Mendelsohn J, Baselga J (2003) Status of epidermal growth factor receptor antagonists in the biology and treatment of cancer. J Clin Oncol 21: 2787–2799. 12860957 10.1200/JCO.2003.01.504

[8] Douillard JY, Siena S, Cassidy J, Tabernero J, Burkes R, et al (2010) Randomized, phase III trial of panitumumab with infusional fluorouracil, leucovorin, and oxaliplatin (FOLFOX4) versus FOLFOX4 alone as first-line treatment in patients with previously untreated metastatic colorectal cancer: The PRIME study. J Clin Oncol 28: 4697–4705. 20921465 10.1200/JCO.2009.27.4860

[9] Van Cutsem E, Kohne CH, Lang I, Folprecht G, Nowacki MP, et al (2011) Cetuximab plus irinotecan, fluorouracil, and leucovorin as first-line treatment for metastatic colorectal cancer: Updated analysis of overall survival according to tumor KRAS and BRAF mutation status. J Clin Oncol 29: 2011–2019. 21502544 10.1200/JCO.2010.33.5091

[10] Bokemeyer C, Bondarenko I, Makhson A, Hartmann JT, Aparicio J, et al (2009) Fluorouracil, leucovorin, and oxaliplatin with and without cetuximab in the first-line treatment of metastatic colorectal cancer. J Clin Oncol 27: 663–671. 19114683 10.1200/JCO.2008.20.8397

[11] Chang DZ, Kumar V, Ma Y, Li K, Kopetz S (2009) Individualized therapies in colorectal cancer: KRAS as a marker for response to EGFR-targeted therapy. J Hematol Oncol 2: 18. 19386128 10.1186/1756-8722-2-18 PMC2686726

[12] Saif MW (2010) Colorectal cancer in review: The role of the EGFR pathway. Expert Opin Investig Drugs 19: 357–369. 10.1517/13543781003593962 20095919

[13] Ozaki N, Ohmuraya M, Hirota M, Ida S, Wang J, et al (2009) Serine protease inhibitor kazal type 1 promotes proliferation of pancreatic cancer cells through the epidermal growth factor receptor. Mol Cancer Res 7: 1572–1581. 19737965 10.1158/1541-7786.MCR-08-0567

[14] Scheving LA (1983) Primary amino acid sequence similarity between human epidermal growth factor-urogastrone, human pancreatic secretory trypsin inhibitor, and members of porcine secretin family. Arch Biochem Biophys 226: 411–413. 6605724 10.1016/0003-9861(83)90309-0

[15] Koskensalo S, Hagstrom J, Louhimo J, Stenman UH, Haglund C (2012) Tumour-associated trypsin inhibitor TATI is a prognostic marker in colorectal cancer. Oncology 82: 234–241. 22508321 10.1159/000336080

[16] Davis NC, Evans EB, Cohen JR, Theile DE (1984) Staging of colorectal cancer. the australian clinico-pathological staging (ACPS) system compared with dukes' system. Dis Colon Rectum 27: 707–713. 6499604 10.1007/BF02554593

[17] Spano JP, Lagorce C, Atlan D, Milano G, Domont J, et al (2005) Impact of EGFR expression on colorectal cancer patient prognosis and survival. Ann Oncol 16: 102–108. 15598946 10.1093/annonc/mdi006

[18] Rego RL, Foster NR, Smyrk TC, Le M, O'Connell MJ, et al (2010) Prognostic effect of activated EGFR expression in human colon carcinomas: Comparison with EGFR status. Br J Cancer 102: 165–172. 19997103 10.1038/sj.bjc.6605473 PMC2813748

[19] McKay JA, Murray LJ, Curran S, Ross VG, Clark C, et al (2002) Evaluation of the epidermal growth factor receptor (EGFR) in colorectal tumours and lymph node metastases. Eur J Cancer 38: 2258–2264. 12441262 10.1016/s0959-8049(02)00234-4

[20] Giralt J, de las Heras M, Cerezo L, Eraso A, Hermosilla E, et al (2005) The expression of epidermal growth factor receptor results in a worse prognosis for patients with rectal cancer treated with preoperative radiotherapy: A multicenter, retrospective analysis. Radiother Oncol 74: 101–108. 15816107 10.1016/j.radonc.2004.12.021

[21] Molaei M, Pejhan S, Nayer BN, Moradi A, Ghiasi S, et al (2009) Human epidermal growth factor receptor-2 family in colorectal adenocarcinoma: Correlation with survival and clinicopathological findings. Eur J Gastroenterol Hepatol 21: 289–293. 19279475 10.1097/MEG.0b013e32830b82ba

[22] Deng Y, Kurland BF, Wang J, Bi J, Li W, et al (2009) High epidermal growth factor receptor expression in metastatic colorectal cancer lymph nodes may be more prognostic of poor survival than in primary tumor. Am J Clin Oncol 32: 245–252. 19451802 10.1097/COC.0b013e3181891326

[23] Galizia G, Lieto E, Ferraraccio F, De Vita F, Castellano P, et al (2006) Prognostic significance of epidermal growth factor receptor expression in colon cancer patients undergoing curative surgery. Ann Surg Oncol 13: 823–835. 16614884 10.1245/ASO.2006.05.052

[24] Resnick MB, Routhier J, Konkin T, Sabo E, Pricolo VE (2004) Epidermal growth factor receptor, c-MET, beta-catenin, and p53 expression as prognostic indicators in stage II colon cancer: A tissue microarray study. Clin Cancer Res 10: 3069–3075. 15131045 10.1158/1078-0432.ccr-03-0462

[25] Fernebro E, Bendahl PO, Dictor M, Persson A, Ferno M, et al (2004) Immunohistochemical patterns in rectal cancer: Application of tissue microarray with prognostic correlations. Int J Cancer 111: 921–928. 15300804 10.1002/ijc.20229

[26] Yasuda H, Tanaka K, Saigusa S, Toiyama Y, Koike Y, et al (2009) Elevated CD133, but not VEGF or EGFR, as a predictive marker of distant recurrence after preoperative chemoradiotherapy in rectal cancer. Oncol Rep 22: 709–717. 19724847 10.3892/or_00000491

[27] Debucquoy A, Goethals L, Libbrecht L, Perneel C, Geboes K, et al (2009) Molecular and clinico-pathological markers in rectal cancer: A tissue micro-array study. Int J Colorectal Dis 24: 129–138. 19050903 10.1007/s00384-008-0608-8 PMC2745734

[28] Chung KY, Shia J, Kemeny NE, Shah M, Schwartz GK, et al (2005) Cetuximab shows activity in colorectal cancer patients with tumors that do not express the epidermal growth factor receptor by immunohistochemistry. J Clin Oncol 23: 1803–1810. 15677699 10.1200/JCO.2005.08.037

[29] Bibeau F, Boissiere-Michot F, Sabourin JC, Gourgou-Bourgade S, Radal M, et al (2006) Assessment of epidermal growth factor receptor (EGFR) expression in primary colorectal carcinomas and their related metastases on tissue sections and tissue microarray. Virchows Arch 449: 281–287. 16865406 10.1007/s00428-006-0247-9 PMC1888717

[30] Saltz LB, Meropol NJ, Loehrer PJ S, Needle MN, Kopit J, et al (2004) Phase II trial of cetuximab in patients with refractory colorectal cancer that expresses the epidermal growth factor receptor. J Clin Oncol 22: 1201–1208. 14993230 10.1200/JCO.2004.10.182

[31] Febbo PG, Ladanyi M, Aldape KD, De Marzo AM, Hammond ME, et al (2011) NCCN task force report: Evaluating the clinical utility of tumor markers in oncology. J Natl Compr Canc Netw 9 Suppl 5: S1–32; quiz S33. 10.6004/jnccn.2011.0137 22138009

[32] Imamura Y, Morikawa T, Liao X, Lochhead P, Kuchiba A, et al (2012) Specific mutations in KRAS codons 12 and 13, and patient prognosis in 1075 BRAF wild-type colorectal cancers. Clin Cancer Res 18: 4753–4763. 22753589 10.1158/1078-0432.CCR-11-3210 PMC3624899

[33] Eklof V, Wikberg ML, Edin S, Dahlin AM, Jonsson BA, et al (2013) The prognostic role of KRAS, BRAF, PIK3CA and PTEN in colorectal cancer. Br J Cancer 108: 2153–2163. 23660947 10.1038/bjc.2013.212 PMC3670497

[34] Ålgars A, Lintunen M, Carpen O, Ristamaki R, Sundstrom J (2011) EGFR gene copy number assessment from areas with highest EGFR expression predicts response to anti-EGFR therapy in colorectal cancer. Br J Cancer 105: 255–262. 21694725 10.1038/bjc.2011.223 PMC3142805

[35] Samuels Y, Wang Z, Bardelli A, Silliman N, Ptak J, et al (2004) High frequency of mutations of the PIK3CA gene in human cancers. Science 304: 554. 15016963 10.1126/science.1096502

[36] Liao X, Lochhead P, Nishihara R, Morikawa T, Kuchiba A, et al (2012) Aspirin use, tumor PIK3CA mutation, and colorectal-cancer survival. N Engl J Med 367: 1596–1606. 23094721 10.1056/NEJMoa1207756 PMC3532946

[37] Wiksten JP, Lundin J, Nordling S, Kokkola A, Stenman UH, et al (2005) High tissue expression of tumour-associated trypsin inhibitor (TATI) associates with a more favourable prognosis in gastric cancer. Histopathology 46: 380–388. 15810949 10.1111/j.1365-2559.2005.02073.x

[38] Paju A, Stenman UH (2006) Biochemistry and clinical role of trypsinogens and pancreatic secretory trypsin inhibitor. Crit Rev Clin Lab Sci 43: 103–142. 16517420 10.1080/10408360500523852

